# Biological Effects of Cyclin-Dependent Kinase Inhibitors Ribociclib, Palbociclib and Abemaciclib on Breast Cancer Bone Microenvironment

**DOI:** 10.3390/ijms23052477

**Published:** 2022-02-24

**Authors:** Michele Iuliani, Sonia Simonetti, Giulia Ribelli, Andrea Napolitano, Umile Giuseppe Longo, Bruno Vincenzi, Paolo Orsaria, Vincenzo Denaro, Giuseppe Tonini, Daniele Santini, Pantano Francesco

**Affiliations:** 1 Department of Medical Oncology, Campus Bio-Medico University of Rome, 00128 Rome, Italy; s.simonetti@unicampus.it (S.S.); ribelli.giuly@live.it (G.R.); a.napolitano@unicampus.it (A.N.); b.vincenzi@policlinicocampus.it (B.V.); g.tonini@policlinicocampus.it (G.T.); d.santini@policlinicocampus.it (D.S.); f.pantano@policlinicocampus.it (P.F.); 2 Royal Marsden Hospital NHS Trust, London SW3 6JJ, UK; 3 Department of Orthopedic and Trauma Surgery, Campus Bio-Medico University of Rome, 00128 Rome, Italy; g.longo@policlinicocampus.it (U.G.L.); v.denaro@policlinicocampus.it (V.D.); 4 Department of Breast Surgery, Campus Bio-Medico University Hospital of Rome, 00128 Rome, Italy; p.orsaria@policlinicocampus.it

**Keywords:** CDK4/6 inhibitors, breast cancer, bone tumor microenvironment, osteoclasts, osteoblasts

## Abstract

The CDK4/6 inhibitors (CDKi) palbociclib, ribociclib, and abemaciclib are currently approved in combination with anti-estrogen therapy for the treatment of advanced and/or metastatic hormone receptor-positive/HER2-neu-negative breast cancer patients. Given the high incidence of bone metastases in this population, we investigated and compared the potential effects of palbociclib, ribociclib, and abemaciclib on the breast cancer bone microenvironment. Primary osteoclasts (OCs) and osteoblasts (OBs) were obtained from human monocyte and mesenchymal stem cells, respectively. OC function was evaluated by tartrate-resistant acid phosphatase assay and real-time PCR; OB activity was assessed by an alizarin red assay. OB/breast cancer co-culture models were generated via the seeding of MCF-7 cells on a layer of OBs, and tumor cell proliferation was analyzed using flow cytometry. Here, we showed that ribociclib, palbociclib, and abemaciclib exerted similar inhibitory effects on the OC differentiation and expression of bone resorption markers without affecting OC viability. On the other hand, the three CDKi did not affect the ability of OB to produce bone matrix, even if the higher doses of palbociclib and abemaciclib reduced the OB viability. In OB/MCF-7 co-culture models, palbociclib demonstrated a lower anti-tumor effect than ribociclib and abemaciclib. Overall, our results revealed the direct effects of CDKi on the tumor bone microenvironment, highlighting differences potentially relevant for clinical practice.

## 1. Introduction

Cyclin-dependent kinases-4 and -6 proteins (CDK4/6) regulate the G1 restriction cell-cycle checkpoint that guards genomic integrity by preventing chromosome duplication until the necessary proteins are assembled [1,2,3]. CDK4/6 proteins activate D-type cyclin [4], leading to the phosphorylation of retinoblastoma (pRb) protein and subsequently E2F phosphorylation, which in turn induces the transcription of cell-cycle genes (e.g., cyclins A and E) as well as transcription-independent functions [1,4,5]. Multiple generations of CDK inhibitors binding to the ATP-binding cleft of CDK enzymes have been created [3,6,7,8]. The last generation of CDK4/6 inhibitors (CDKi) includes palbociclib (PD 0332991), ribociclib (LEE011), and abemaciclib (LY2835219), which are currently approved for the treatment of advanced and/or metastatic hormone receptor-positive/HER2-neu-negative (HR+/HER2−) breast cancer in combination with hormonal therapy. These combinations have significantly improved clinical outcomes when compared to anti-estrogen monotherapy, as demonstrated by the randomized phase III trials PALOMA, MONALEESA, and MONARCH [9,10,11,12,13,14,15,16]. Although these drugs have reported comparable clinical benefits, there are unique differences in their substrate selectivity and pharmacodynamics [17,18,19] that could explain the varying differences observed in certain clinical settings. For example, abemaciclib is the only CDKi also approved as a monotherapy in the metastatic setting, and promising data support its use in the adjuvant setting. Moreover, there are also differences among the three CDKi in terms of their ability to penetrate the blood–brain barrier, their side effects, and the dosing schedules that need to be taken into account to optimize the selection of treatment for individual patients. Intriguingly, subgroup analyses in patients with bone-only disease suggest that all three CDKi may help reduce disease progression in the bone metastatic sites [15,20,21]. Nevertheless, no data concerning the direct or indirect effect of these agents on the bone microenvironment are available.

Bone is the most common site of breast cancer metastases and maintaining bone health is crucial in breast cancer patients undergoing endocrine therapy, which can adversely affect the skeleton. For these reasons, here we performed a comparative analysis on the effects of ribociclib, palbociclib, and abemaciclib on the bone microenvironment, evaluating of osteoclast (OC) and osteoblast (OB) function. Moreover, the anti-tumor activity of these compounds was investigated in in vitro “cell–cell contact” co-culture models of breast cancer cells and OBs that mimic the bone tumor microenvironment.

## 2. Results

### 2.1. Comparative Effect of CDKi on Breast Cancer Cells

**Figure 1:**
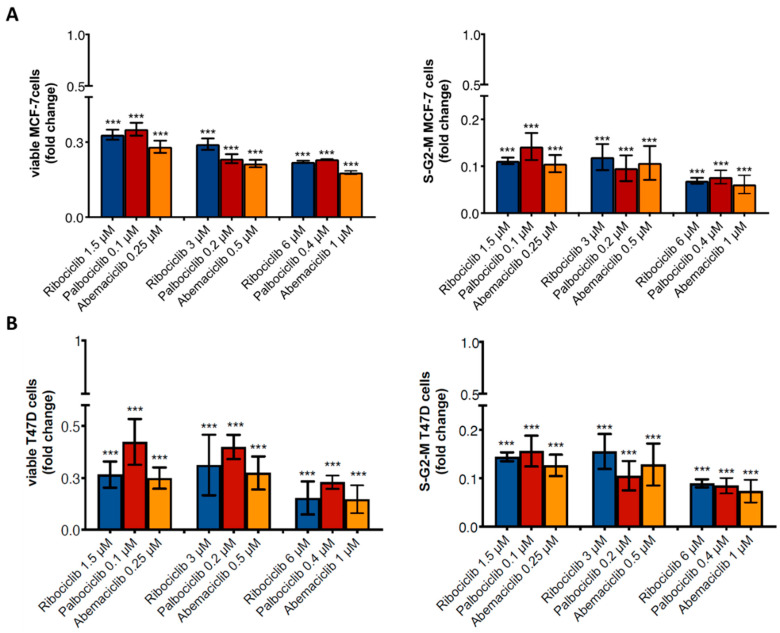
Anti-tumor effect of CDKi on MCF-7 and T47D breast cancer cells. Analysis of MCF-7 (**A**) and T47D (**B**) viability and proliferation (cells in S-G2-M phases) after CDKi treatment by flow cytometry. *p*-value was calculated comparing MCF-7 cells treated vs. untreated (control = 1). *** *p* < 0.0001. Number of replicates: 4; statistical test: Student *t*-test.

Hormone-sensitive breast cancer cells, MCF-7 and T47D, were treated with clinically relevant concentrations of ribociclib (1.5 μM, 3 μM, 6 μM), palbociclib (0.1 μM, 0.2 μM, 0.4 μM), and abemaciclib (0.25 μM, 0.5 μM, 1 μM) for 7 days (22–24). Data showed that CDKi exerted a similar effect on MCF-7 and T47D cells, reducing cell viability compared to the control (*p* < 0.0001) (Figure 1A). In addition, cell cycle analyses revealed that these agents had a similar ability to decrease the number of cells in S-G2-M phases (*p* < 0.0001) (Figure 1B). These data suggest that the choice of concentrations are appropriate for performing a comparative analysis of the three drugs, comparing their low, median, and high doses.

### 2.2. Comparative Effect of CDKi on Bone Cells

**Figure 2:**
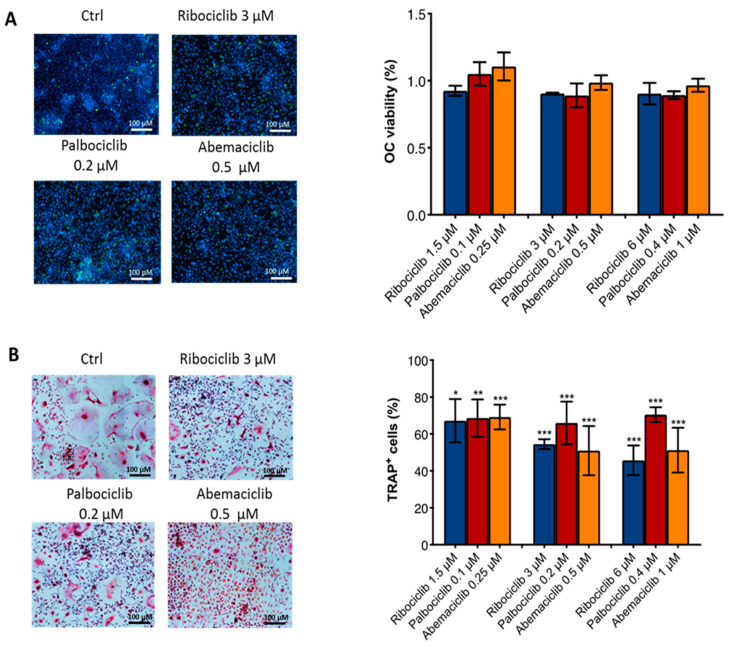
Effect of CDKi on OC viability and differentiation. (**A**) Representative images of OC viability after CDKi treatment at median concentrations (live cells (blue), dead cells (green)) and comparative analysis of OC viability at all drugs concentrations. (**B**) Representative images of TRAP assay after CDKi treatment at median concentrations and comparative analysis of TRAP-positive cells at all drugs concentrations. *p*-value was calculated comparing OCs treated vs. untreated (control = 1 or control = 100). (ribociclib 1.5 μM, *p* = 0.01, ribociclib 3 μM, *p* < 0.0001, ribociclib 6 μM, *p* < 0.0001; palbociclib 0.1 μM, *p* = 0.0005, palbociclib 0.2 μM, *p* < 0.0001, palbociclib 0.4 μM, *p* < 0.0001; abemaciclib 0.25 μM, *p* < 0.0001, abemaciclib 0.5 μM, *p* < 0.0001, abemaciclib 1 μM, *p* < 0.0001) * *p* < 0.01, ** *p* < 0.001, *** *p* < 0.0001. Number of replicates: 5; statistical test: Student *t*-test.

To evaluate the potential effect of the three CDKi on the bone microenvironment, we analyzed the viability and differentiation of primary human OCs and Obs after treatments with ribociclib, palbociclib, and abemaciclib. Our results showed that CDKi did not affect the OC viability (Figure 2A). Intriguingly, the three agents inhibited OC differentiation at all doses analyzed (Figure 2B).

**Figure 3:**
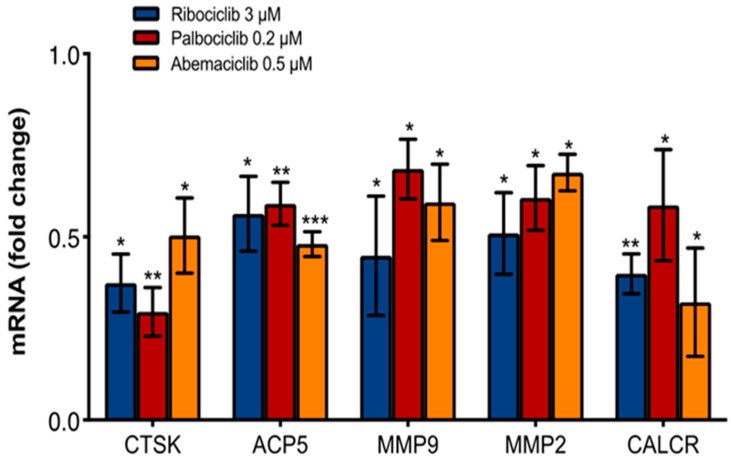
Effect of CDKi on bone resorption markers. Reduction in CTSK (ribociclib 3 μM, *p* = 0.001; palbociclib 0.2 μM, *p* = 0.0004; abemaciclib 0.5 μM, *p* = 0.008), ACP5 (ribociclib 3 μM, *p* = 0.01; palbociclib 0.02 μM, *p* = 0.0004; abemaciclib 0.5 μM, *p* < 0.0001), MMP9 (ribociclib 3 μM, *p* = 0.02; palbociclib 0.2 μM, *p* = 0.02; abemaciclib 0.5 μM, *p* = 0.02), MMP2 (ribociclib 3 μM, *p* = 0.01; palbociclib 0.2 μM, *p* = 0.01; abemaciclib 0.5 μM, *p* = 0.002) and CALCR (ribociclib 3 μM, *p* = 0.0001; palbociclib 0.2 μM, *p* = 0.04; abemaciclib 0.5 μM, *p* = 0.01) gene expression after CDKi treatment at median concentrations. *p*-value was calculated comparing OCs treated vs. untreated (control = 1). * *p* < 0.01, ** *p* < 0.001, *** *p* < 0.0001. Number of replicates: 3; statistical test: Student *t*-test.

In addition, CDKi were able to reduce the mRNA expression of the main bone resorption genes Cathepsin K (CTSK), Acid Phosphatase 5, Tartrate Resistant (ACP5), Metalloproteinase 9 (MMP9), Metalloproteinase 2 (MMP2), and Calcitonin Receptor (CALCR) (at the median concentration (Figure 3)). These data demonstrated that all three agents had a similar inhibitory effect on the OC differentiation and activity.

**Figure 4:**
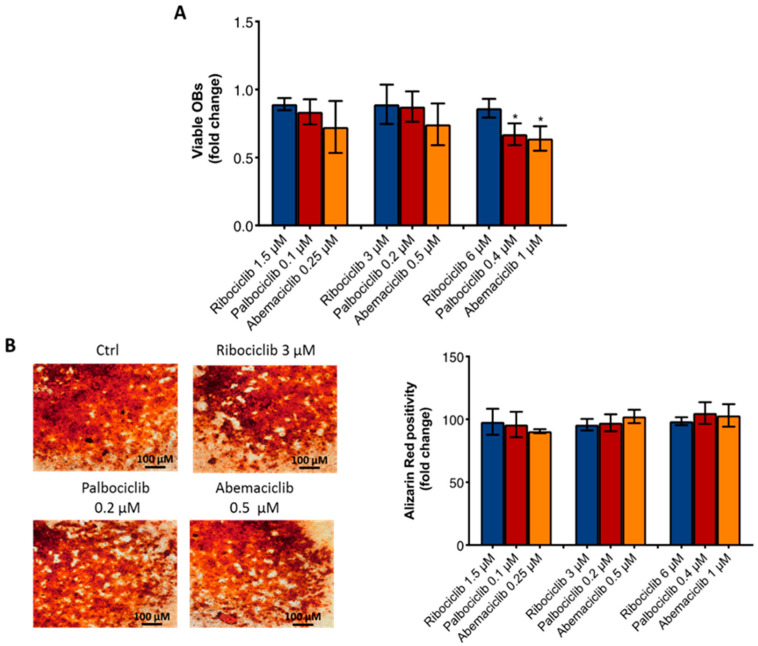
Effect of CDKi on OB viability and activity. (**A**) Comparative analysis of OB viability at all drug concentrations. Cytotoxicity was observed with palbociclib (0.4 μM) (*p* = 0.004) and abemaciclib (1 μM) (*p* = 0.005) (**B**) Representative images of Alizarin Red assay after CDKi treatment at median concentrations and comparative analysis of Alizarin Red positivity at all drug concentrations. *p*-value was calculated comparing OBs treated vs. untreated (control = 1). * *p* < 0.01. Number of replicates: 5; statistical test: Student *t*-test.

The viability analysis of OBs after CDKi treatment revealed that only palbociclib (0.4 μM) and abemaciclib (1 μM) significantly reduced OB viability by 33% and 36%, respectively, but no significant differences were found when comparing the effect of the agents at low, median, and high doses (Figure 4A). Finally, the ability of OB to produce bone matrix was not affected by the CDKi treatment (Figure 4B).

### 2.3. Comparative Effect of CDKi on Bone Tumor Microenvironment

**Figure 5:**
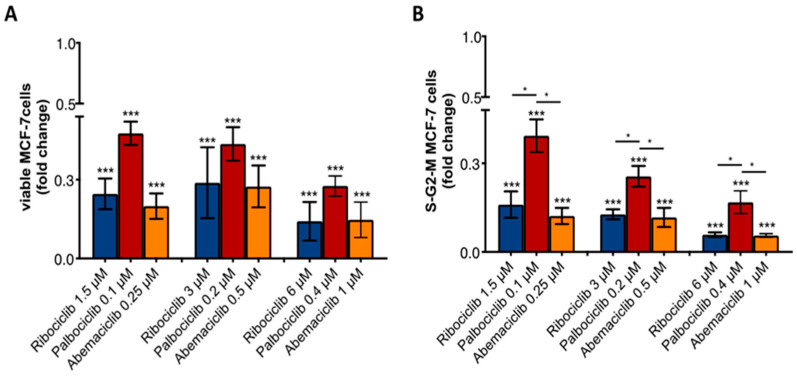
Anti-tumor effect of CDKi on MCF-7 breast cancer cells co-cultured with OBs. (**A**) Analysis of MCF-7 viability and (**B**) proliferation (cells in S-G2-M phases) after CDKi treatment by flow cytometry. *p*-value was calculated comparing MCF-7 cells treated vs. untreated (control = 1) and comparing CDKi agents each other. Ribociclib 1.5 μM vs. palbociclib 0.1 μM *p*= 0.03; ribociclib 3 μM vs. palbociclib 0.2 μM *p*= 0.02; ribociclib 6 μM vs. palbociclib 0.4 μM *p* = 0.048; abemaciclib 0.25 μM vs. palbociclib 0.1 μM *p*= 0.01; abemaciclib 0.5 μM vs. palbociclib 0.2 μM *p*= 0.040; abemaciclib 1 μM vs. palbociclib 0.4 μM *p* = 0.049. * *p* < 0.01, *** *p* < 0.0001. Number of replicates: 4; statistical test: Student *t*-test.

To investigate the effect of CDKi in a bone tumor microenvironment, we set up a direct cell–cell contact co-culture between OBs and MCF-7 cells. As reported in Figure 5A, the three drugs significantly reduced cell viability and proliferation compared to the control (*p* < 0.0001). Intriguingly, comparative analysis showed that ribociclib and abemaciclib exerted a higher anti-proliferative effect in terms of number of cells in S-G2-M phases, compared to palbociclib (Figure 5B). These data suggest that the presence of OBs reduced the anti-tumor effect of palbociclib, but did not influence the activity of ribociclib and abemaciclib, at clinically relevant doses.

## 3. Discussion

Besides the overall similarities of the three approved CDK4/6 inhibitors in terms of class activity and improvements in clinical outcomes, there are important pharmacological differences between them. The different mean half-life of ribociclib, palbociclib, and abemaciclib result in differences in dosing schedules and serum plasma concentrations. For this purpose, based on the mean plasma concentrations at steady state reported in phase I pharmacokinetics studies [22,23,24], we preliminarily selected the proper dosage ranges for each compound to perform comparative analyses. The treatment of breast cancer cells, MCF-7 and T47D, at the chosen doses, showed a similar anti-tumor effect of the three drugs, confirming that the selected concentrations were appropriate for comparative in vitro experiments. A previous study compared the transcriptional, proteomic, and phenotypic changes induced by palbociclib, ribociclib, and abemaciclib on breast cancer cells, finding significant differences among the three agents [25]. However, differently from our study, the authors used equivalent doses of drugs without taking into account their specific pharmacokinetics.

Despite the fact that CDKi are approved in metastatic settings and that breast cancer has a high osteotropism, no data about the direct and indirect effects of these drugs on bone are available. Thus, we investigated if ribociclib, palbiciclib, and abemaciclib influenced the bone microenvironment, modulating OC and OB viability and activity. To mimic in vivo pharmacological estrogen deprivation, we used charcoal-stripped serum.

Primary human models of OCs and Obs were generated to resemble the physiological bone. In this regard, the choice of these models and the use of appropriate drug concentrations could provide a clinically relevant picture of the impact of CDKi on the skeleton. Our data showed that the three CDKi exerted an inhibitory effect on OC differentiation without affecting their viability. In addition, drug treatment significantly reduced the expression of the main genes involved in bone resorption, such as Cathepsin K, TRAP, MMP-9, MMP-2, and Calcitonin Receptor. This inhibitory effect did not differ across the three compounds, suggesting that the doses tested had a comparable activity in suppressing osteoclastogenesis. These results are in accordance with previous data showing G1 arrest in OC precursors and an inhibition of the OC progenitor pool expansion after CDKi treatment [26].

In contrast, the highest clinical dose of palbociclib and abemaciclib significantly reduced OB viability, while ribociclib did not show any cytotoxic effect. In the context of estrogen deprivation therapy associated with an increased risk of bone loss, investigating the direct effect of these agents on OBs could be clinically relevant. Our results suggest that ribociclib may protect bone integrity more than the other two compounds, maintaining OB viability.

Significant differences among the drugs were also observed in OB/cancer cells co-culture models in terms of their anti-tumor activity. Although all compounds significantly reduced breast cancer cell proliferation in the bone microenvironment compared to the control, ribociclib and abemaciclib showed a higher efficacy than palbociclib. These different biological effects could be explained by the different kinome selectivity profiles of the three compounds. Indeed, biochemical interaction analyses revealed that all drugs are highly selective for CDK4/6, but abemaciclib binds multiple other kinases (18). In addition, palbociclib has a similar potency against cyclin D1/CDK4 and cyclin D2/CDK6 [27], while abemaciclib and ribociclib have greater potency against CDK4 than CDK 6 [28,29]. The different spectrum and degree of interactions of the three agents could influence their clinical performance. In addition, increasing evidence shows a crucial role of OBs in promoting breast cancer survival and progression in the bone. In particular, osteoblast-lineage cells express high levels of -X-C motif chemokine ligand 12 (CXCL12), receptor activator of nuclear factor κ-B ligand (RANKL), interleukin-6, Vascular-Endothelial Growth Factor (VEGF), Transforming growth factor-beta (TGF-β), and platelet-derived growth factor receptors (PDGFs). All of these proteins have the ability to activate the E2F-pRb pathway and induce the proliferation of breast cancer cells [30,31,32] As a result, only ribociclib and abemaciclib, given at clinically relevant doses, showed the same efficacy against cancer cells grown in the bone tumor microenvironment and as a monocolture.

## 4. Materials and Methods

### 4.1. Drug Treatments

Drugs were used at the following concentrations: ribociclib 1.5 μM, 3 μM, 6 μM; palbociclib 0.1 μM, 0.2 μM, 0.4 μM; abemaciclib 0.25 μM, 0.5 μM, 1 μM. All treatments were performed in culture media supplemented with 10% of charcoal-stripped serum to simulate estrogen deprivation.

### 4.2. Primary Human Osteoclasts

Primary human OCs were isolated from the human peripheral blood mononuclear cells (PBMCs) of healthy blood donors, as previously described [33]. The procedure was approved by the Ethical Committee of Campus Bio-Medico University of Rome (Prot 21/15 OSS) in accordance with the Declaration of Helsinki principles. Briefly, CD14+ monocytes were differentiated in OCLs in the presence of 25 ng/mL macrophage-colony stimulating factor (M-CSF) and 50 ng/mL receptor activator of Nuclear Factor kappa-B ligand (RANKL) (R&D Systems, Minneapolis, MN, USA) for 12 days. Ribociclib, palbociclib, and abemaciclib were added, every 3 days, to the culture medium for 7 days (from day 5 to day 12). OC viability was performed using the ReadyProbes™ Cell Viability Imaging Kit, Blue/Green (Thermo Fisher Scientific, Waltham, MA, USA) according to the manufacturer’s protocol. OC differentiation was evaluated by acid phosphatase and tartrate-resistant acid phosphatase (TRAP) assay (Sigma Aldrich, St. Louis, MO, USA) at the end of the differentiation protocol, following manufacturer’s instructions.

### 4.3. Primary Human Osteoblasts

Human primary OBs were obtained from bone marrow samples of healthy patients undergoing total hip replacement at Policlinico Campus Bio-Medico. The procedure was approved by the Ethical Committee of the Campus Bio-Medico University of Rome and informed consent from patients was collected in accordance with the Declaration of Helsinki principles (Prot 21/15 OSS). Bone marrow mesenchymal stem cells (BM-MSCs) were isolated as previously described [34]. OB differentiation was achieved by culturing BM-MSCs at an initial density of 5 × 10^4^ in 24-well plates while adding 10 mM beta-glycerophosphate (Sigma Aldrich, St. Louis, MO, USA), 50 μM ascorbic acid (Sigma Aldrich, St. Louis, MO, USA), and 100 nM dexamethasone (Sigma Aldrich, St. Louis, MO, USA) to the culture medium for 28 days. OBs were treated with ribociclib, palbociclib, and abemaciclib for 7 days (from day 21 to day 28). OB viability was evaluated by flow cytometry using Fixable Viability Dye conjugated with eFluor780 fluorochrome (eBioscience-Thermo Fisher Scientific, Waltham, MA, USA). OB activity was analyzed by Alizarin Red staining (Sigma Aldrich, St. Louis, MO, USA) following the manufacturer’s instructions.

### 4.4. Breast Cancer Cells Cultures

Hormone-sensitive breast cancer cells MCF-7 were purchased from the American Type Culture Collection (ATCC, Manassas, VA, USA). MCF-7 cells were grown in Eagle’s Minimum Essential Medium with 10% of Fetal Bovine Serum (Gibco-Thermo Fisher Scientific, Waltham, MA, USA), supplemented with 100 units/liter Penicillin, 100 μg/mL Streptomycin (EuroClone, Milano, Italy), 2 mM Glutamine (EuroClone, Milano, Italy), and 0.01 mg/mL human recombinant insulin (Sigma Aldrich). MCF-7 cells (50 × 10^4^) were cultured alone or on an OB layer in 24-well plates and treated or not with Ribociclib, palbociclib, and abemaciclib. After 7 days, MCF-7 cells and OB cell cycle analyses were performed using flow cytometry.

### 4.5. Cell Cycle Analysis

**Figure 6:**
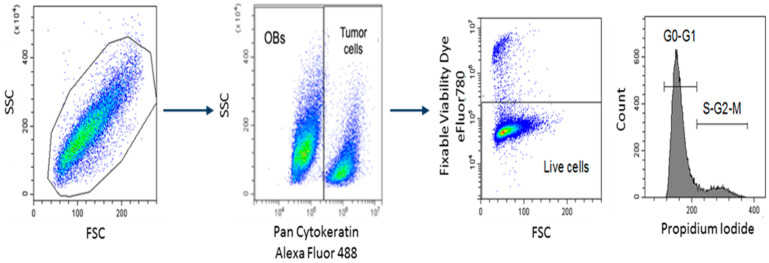
Gating strategy cell cycle analysis of MCF-7 in co-culture with OBs. Cells are stained with anti-Pan Cytokeratin Alexa Fluor 488 to discriminate MCF-7 from OBs and with Propidium Iodide for cell cycle analysis. Death cells were excluded using Fixable Viability Dye eFluor780. The gating strategy was based on Forward and Side scatter.

The cell cycle was analyzed using the following gaiting strategy (Figure 6) [35]. Briefly, cells were fixed and permeabilized with Foxp3/Transcription Factor Staining Buffer Set (eBioscience-Thermo Fisher Scientific, Waltham, MA, USA) for intracellular staining with anti-Pan Cytokeratin Alexa Fluor 488 (clone AE1/AE3 eBioscience-Thermo Fisher Scientific, Waltham, MA, USA), anti-Ki67-APC (clone 20Raj1 eBioscience-Thermo Fisher Scientific, Waltham, MA, USA), and a Propidium Iodure (PI) solution (50 μg/mL PI+ 40 ng/mL RNAseA+ 0.1% of Triton) ((Sigma Aldrich, St. Louis, MO, USA). Dead cell exclusion was performed with Fixable Viability Dye conjugated with eFluor780 fluorochrome (eBioscience-Thermo Fisher Scientific, Waltham, MA, USA). Samples were analyzed by CytoFlex instrument (Beckman Coulter, Brea, CA, USA) using the CytExpert Software, v.2.1.

### 4.6. Real Time PCR

Total RNA was extracted by Trizol reagent (Invitrogen - Thermo Fisher Scientific, Waltham, MA, USA) according to the manufacturer’s instructions. cDNA was produced using the High Capacity cDNA Reverse Transcription kit (Applied Biosystems-Thermo Fisher Scientific, Waltham, MA, USA) according to the manufacturer’s instructions. mRNA levels were measured by quantitative real-time polymerase chain reaction (qRT-PCR) using the TaqMan Gene Expression Assays in 7900HT Real-Time PCR System (Applied Biosystems-Thermo Fisher Scientific, Waltham, MA, USA). The CTSK (Hs00166156_m1), ACP5 (Hs00356261_m1), MMP9 (Hs00234579_m1), MMP2 (Hs001548727_m1), and CALCR (Hs001016882_m1) expression levels were normalized to the endogenous housekeeping gene Glucuronidase Beta (GUSβ) (Hs99999908_m1).

### 4.7. Statistical Analysis

Data were analyzed using the Student *t*-test and one-way ANOVA followed by Tukey’s multiple comparison tests. The graphics processing and statistical tests were performed using the program GraphPad Prism (San Diego, CA, USA).

## 5. Conclusions

Taken together, our data reveal the novel direct effects of ribociclib, palbociclib, and abemaciclib, at clinically relevant concentrations, on the bone tumor microenvironment, along with the similarities and differences among the three agents. All compounds exerted a similar inhibitory effect on OC differentiation, modulating the main OC marker genes without affecting the OC viability. On the other hand, the three CDKi showed a different impact on OB viability. Indeed, the highest concentrations of palbociclib and abemaciclib were cytotoxic on OBs, while ribociclib treatment preserved bone integrity at all clinical doses. Maintaining bone health in breast cancer patients is fundamental, since they often experience multifactorial bone loss due to both the high frequency of bone metastasis as well as the anti-cancer treatments received. In our OB/tumor cells co-culture, we observed a lower anti-tumor effect of palbociclib compared to ribociclib and abemaciclib. In this model that mimics the bone tumor microenvironment, the proliferative signals of OBs could modulate the CDK profile of tumor cells and influence the effect of CDKi. However, the CDKi effects observed on bone cells and in bone/tumor co-cultures could be influenced by treatment with anti-resorptive agents, such as zoledronic acid or denosumab. In this regard, it could be worth investigating the specific contribution of each agent in modulating tumor bone milieu. Moreover, the evaluation of the potential activity of CDKi in other components of the tumor microenvironment, such as in immune cells, could be relevant for optimizing their use in clinical practice.

## Data Availability

Not applicable.
